# Analytical Determination of Squalene in Extra Virgin Olive Oil and Olive Processing By-Products, and Its Valorization as an Ingredient in Functional Food—A Critical Review

**DOI:** 10.3390/molecules29215201

**Published:** 2024-11-03

**Authors:** Laura Barp, Ana Miklavčič Višnjevec, Sabrina Moret

**Affiliations:** 1 Department of Agri-Food, Environmental and Animal Sciences, University of Udine, 33100 Udine, Italy; sabrina.moret@uniud.it; 2 Faculty of Mathematics, Natural Sciences and Information Technologies, University of Primorska, Glagoljaška 8, 6000 Koper, Slovenia; ana-miklavcic@famnit.upr.si

**Keywords:** analysis of squalene, extra virgin olive oil, olive oil processing by-products

## Abstract

Squalene is a bioactive compound with significant health benefits, predominantly found in extra virgin olive oil (EVOO) and its processing by-products. This critical review explores the analytical determination of squalene in EVOO and various by-products from olive oil production, highlighting its potential as a valuable ingredient in functional foods. An overview of existing analytical methods is provided, focusing on different approaches to sample preparation before analytical determination, evaluating their effectiveness in quantifying squalene concentrations. Studies not primarily centered on analytical methodologies or squalene quantification were excluded. A critical gap identified is the absence of an official method for squalene determination, which hinders comparability and standardization across studies, underscoring the importance of developing a reliable, standardized method to ensure accurate quantification. The valorization of squalene involves advocating for its extraction from olive oil processing by-products to enhance sustainability in the olive oil industry. By recovering squalene, the industry can not only reduce waste but also enhance functional food products with this health-promoting compound. Additionally, there is a need for economically sustainable and environmentally friendly extraction techniques that can be scaled up for industrial application, thus contributing to a circular economy within the olive oil sector.

## 1. Introduction

According to the Food and Agriculture Organization (FAO) of the United Nations, functional foods are foods that contain, in addition to nutrients, bioactive components that may be beneficial to health [1]. Based on this definition, functional foods include healthy foods in their original form (e.g., extra virgin olive oil - EVOO), as well as fortified and enriched foods with added bioactive compounds [2].

Due to its production method, which prevents the deterioration of natural bioactive compounds, EVOO represents the highest quality category of olive oils. Many authors attribute the health properties of EVOO to the high presence of oleic acid (a monounsaturated fatty acid), phenolic components, and tocopherols (vitamin E). All of these bioactive compounds have been recognized as able to reduce the risk of cardiovascular diseases and inflammatory processes associated with various diseases [3,4].

As proof of the health properties of EVOO, four health claims applicable to this product have been approved by the European Food Safety Authority (EFSA) in recent years [5,6]. Two of these claims refer to the health benefits of replacing saturated fatty acids with unsaturated ones found in high amounts in olive oils due to the high presence (66–78%) of oleic acid. Another claim highlights the role of vitamin E in preventing oxidative stress, while the final claim, specific to olive oils, states that “polyphenols in olive oil contribute to the protection of blood lipids from oxidative stress”. This claim has been authorized based on human studies showing significantly reduced levels of oxidized low-density lipoproteins in plasma after the consumption of virgin olive oil, and it can only be used for olive oil that contains at least 5 mg of hydroxytyrosol and its derivatives per 20 g of olive oil.

In addition to containing a high content of the bioactive components mentioned above, olive oil also contains a significant amount of squalene. Squalene is a naturally occurring triterpene that has attracted much attention for its health benefits and widespread presence in various plant and animal sources [7,8]. Although EVOO is a particularly rich source of squalene, making it of interest to researchers and the food industry, its high presence in olive oil is not sufficiently valued. This is partly due to the lack of approved health claims for squalene or products containing it, despite its widespread use as a food supplement. In Italy, squalene is authorized as a food supplement with no defined maximum daily intake by the Ministry of Health. The health benefits attributed to squalene include its supposed protective action on DNA, proteins, and lipids against oxidative stress, its ability to defend the epidermis from damage caused by ultraviolet rays, and its anti-cancer and anti-inflammatory properties [9,10]. However, due to insufficient scientific evidence, the EFSA has rejected the claim that squalene can protect DNA, proteins, and lipids from oxidative stress [11,12]. For a health claim to be successful, supporting documentation must include detailed information on the characteristics of the food or constituent in question, factors that may influence the claimed effect (such as composition, physical and chemical characteristics, manufacturing process, and stability), and a validated measurement method [13].

The first part of this critical review summarizes the main positive health effects highlighted in the recent literature and will explore the importance of EVOO and olive oil by-products as a source of squalene in the diet. The second part of this review will delve into the analytical methodologies employed to accurately quantify squalene in EVOO and olive processing by-products, with a special focus on sample preparation, which is a critical point for obtaining reliable results, and chromatographic techniques able to enhance the precision and reliability of squalene detection and measurement. This review presents a novel contribution by highlighting the lack of coordination in standardizing the analysis of squalene from olive oil and its by-products. This gap in standardization presents a significant barrier to unlocking the full potential of squalene as a valuable functional ingredient. This perspective not only focuses on improving squalene quantification but also contributes to the broader conversation about sustainability and circular economy in the olive oil industry.

## 2. Methods

This critical review was conducted by performing a search, reviewing articles, and discussing the results. PubMed, Scopus, Science Direct, Web of Science, and Google Scholar were searched to identify relevant studies that align with the objectives of this review. Studies not primarily focused on analytical methodologies or squalene quantification were excluded. The selected studies were summarized and critically commented on, highlighting critical points or gaps, as well as innovative approaches.

## 3. Literature Background on Squalene

### 3.1. Chemical Structure, Physical Properties, and Biosynthesis of Squalene

**Figure 1:**
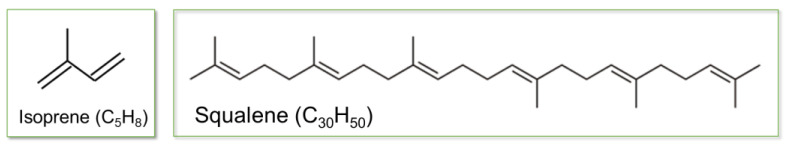
Chemical structure of the basic units of isoprene (C_5_H_8_) and squalene (C_30_H_50_).

Squalene is a hydrocarbon belonging to the class of triterpenes, with the formula C_30_H_50_ (410.73 g/mol) (Figure 1). It was first isolated in 1916 from *Squalus* spp. liver oil, from which it derives its name [7]. It consists of six isoprene units joined in a head-to-tail manner, forming a linear chain. The six double bonds, which are conjugated in a 1.4 pattern along the chain, are in trans structures. Therefore, squalene easily oxidizes and gives off a fishy smell when exposed to air for a long time. It can cyclize to give bicyclic, tetracyclic, and pentacyclic triterpenoid structures. The main forms include 2 sterol-like forms, a coiled form (pentacyclic), and a stretched form [14].

Squalene is a colorless, odorless liquid grease, hydrophobic and insoluble in water, sparingly soluble in alcohol and glacial acetic acid, but freely soluble in ether, petroleum ether, chloroform, acetone, and other non-polar solvents. Its melting and boiling points are −75 °C and 245 °C, respectively [7,15]. The biosynthesis of squalene is a crucial step in the metabolic pathway that leads to the production of sterols, including cholesterol in animals and phytosterols in plants [16]. In animal cells, this process occurs through the mevalonate pathway (MVA pathway), which involves several steps: (i) conversion of acetyl-CoA into 3-hydroxy-3-methylglutaryl-CoA (HMG-CoA) through a series of enzyme-catalyzed reactions, (ii) formation of mevalonate by the enzyme HMG-CoA reductase, (iii) production of isopentenyl pyrophosphate after phosphorylation and decarboxylation of mevalonate, (iv) formation of geranyl pyrophosphate and farnesyl pyrophosphate, (v) synthesis of squalene from head-to-tail condensation of two geranyl pyrophosphate molecules [16,17]. In plants, the biosynthetic pathway of sterols differs slightly from that of animal cells and fungi. Isoprenoids in plants can be synthesized through the MVA pathway in the cytosol, leading to the production of sterols and brassinosteroids, or in mitochondria where side chains of ubiquinone are formed. Alternatively, the non-MVA pathway, located in plastids leads to the synthesis of carotenoids, side chains of chlorophylls, plastoquinones, and isoprenoid-type phytohormones. This pathway begins with the condensation of glyceraldehyde-3-phosphate and pyruvate to form 1-deoxy-D-xylulose-5-phosphate, which serves as a precursor in thiamine and pyridoxol biosynthesis [16,18].

Animal and human studies have shown that nearly 60% of squalene introduced through the diet is absorbed [19]. The unabsorbed squalene is partly excreted in feces and probably partly metabolized by the gut microbiota, but further studies are needed to clarify this pathway [19].

### 3.2. Bioactive Properties of Squalene

Numerous studies have provided support for the various bioactivities of squalene, including antioxidant, anti-inflammatory, anti-cancer, and anti-atherosclerosis properties, both in vivo and in vitro [20]. The role of squalene as an antioxidant is particularly significant as it helps neutralize free radicals, which are unstable molecules that can damage cells, contributing to aging and various diseases. The antioxidant capability of squalene is crucial for maintaining cellular integrity and overall health, reducing oxidative stress, and mitigating the risk of chronic diseases such as cardiovascular disorders [18,20,21]. In addition to its antioxidant activity, when introduced through the diet, squalene’s ability to penetrate the skin quickly and deeply, without leaving a greasy residue, enhances skin hydration and helps protect the skin surface from lipid peroxidation caused by exposure to ultraviolet light and other sources of oxidative damage [20,22].

While squalene serves as a key intermediate in the endogenous production of cholesterol, providing it in the diet of patients with hypercholesterolemia significantly decreases total cholesterol and low-density lipoprotein cholesterol levels, while increasing high-density lipoprotein cholesterol levels [17,18,20]. The mechanism of action is similar to that of statins and is associated with the inhibitory activity exerted by squalene on HMG-CoA reductase in the liver, which reduces the conversion of acetyl-CoA to cholesterol [10]. Based on the meta-analysis conducted by Ibrahim et al. [10], aimed at evaluating if squalene can be effective in preventing cardiovascular diseases (a leading cause of death worldwide), 15 out of 18 animal studies and 1 out of 3 human studies showed positive outcomes.

Squalene is known to reduce neutrophil and monocyte activation while increasing anti-inflammatory enzymes [23]. Due to its anti-inflammatory, immunomodulatory, antioxidant, and antiviral properties, its use in contrasting SARS-CoV-2 infection was investigated. Squalene extracted from pumpkin seeds and encapsulated in a microemulsion for sublingual use has been associated with a significant decrease in mortality and re-hospitalization rates among COVID-19 patients [24]. Additionally, squalene appears to play a crucial role in retinal health, particularly in reducing free radical oxidative damage in rod photoreceptor cells [19,20].

Some studies have suggested that squalene can inhibit the proliferation of cancer cells and enhance the efficacy of certain chemotherapy drugs. However, more research is needed to fully understand these mechanisms and their clinical applications [15,21,25,26]. Additionally, squalene is used in the pharmaceutical sector as a drug delivery agent, an adjuvant in vaccines, a detoxifier, and a skin moisturizer or protector [17,18,26,27,28].

To fully understand the bioactive properties of squalene, the reader is directed to some recent reviews [9,10,19,29].

### 3.3. Sources of Squalene and Dietary Intake

Squalene is naturally synthesized in the human body (1.5 g/day) as an intermediate in the cholesterol biosynthesis pathway [30]. It is a major constituent (12%) of human sebum, and is present in various tissues, contributing to skin hydration and protection [30]. Due to its widespread occurrence and beneficial properties, squalene has been extensively studied and used in industries such as pharmaceuticals, cosmetics, and food [8,31,32].

Its presence in both plant and animal sources makes squalene a versatile compound with significant commercial and health-related potential.

It is widely distributed in nature and can be found in animals, plants, and microorganisms such as yeast, fungi, cyanobacteria, and marine microalgae [7]. In plants, squalene serves multiple functional roles, acting as a precursor for the production of various bioactive compounds and as a nutrient [7].

Historically, squalene was first discovered in shark liver oil, where it can constitute up to 70–80% of the oil and it was the primary source of squalene [30]. However, concerns about preserving marine life and the presence of persistent organic pollutants and heavy metals have limited the use of this natural source of squalene. To meet the growing demand for squalene after restrictions on shark-derived squalene, there is a need to find a low-cost and sustainable alternative for its production.

Some fungi and yeasts produce squalene, which can be extracted for commercial use [31]. Furthermore, in recent years, great progress has been made using microbial strains, both wild and engineered, capable of producing squalene with high productivity [33]. For example, Song et al. [34] applied metabolic engineering to produce squalene from *Bacillus subtilis*.

Based on the literature data [30], squalene concentration in vegetable oils (produced on a large scale in Europe) range from 0 to 0.19 g/kg in sunflower oil, from 0.03 to 0.2 g/kg in soybean oil, from 0.1 to 0.17 g/kg in corn oil [35], and from 1.7 to 4.6 g/kg in olive oil [36]. Reasonable amounts of squalene have also been found in other vegetable oils such as rice bran oil, palm oil, and wheat germ oil [8]. A study carried out on different genotypes of *Amaranthus* sp. reported squalene concentrations from 10.4 to 73.0 g/kg in the oil. This makes this plant the vegetable source with the highest concentration of squalene. Recovering squalene directly from amaranth seeds is an expensive process, but it could be advantageous to recover it from the oil [37].

#### 3.3.1. Squalene in Olive Oils

Based on literature data, virgin olive oil, obtained from the fruit of the olive tree *Olea europaea* L., is one of the richest dietary sources of squalene, containing up to 0.7% by weight. It is surpassed in squalene content by shark liver and amaranth oil (Amaranthus is a pseudo-grain from *Amaranthus* sp.). Olive oil contains up to 300 times more squalene than other vegetable oils and up to 5000 times more than some vegetable foods [20]. The average daily dietary intake of squalene in Mediterranean countries, which follow a diet rich in virgin olive oil, is in the range of 200–400 mg/day, whereas in the United States, intake has been estimated to be around 30 mg/day [20,22,38]. According to Tsimidou et al. [39]., the hydrocarbon fraction of olive oil, primarily composed of squalene, typically contains squalene levels ranging from 2 to 12 g/kg, with an average level of 5 mg/kg. Statistically processing the squalene content of 308 individual data points from 33 bibliographic references, Martínez-Beamonte et al. [8] reported an average level of 4.554 ± 2.095 g/kg. The same authors observed high variability in squalene concentration depending on the olive cultivar. The cultivars with the highest squalene content were Nocellara de Belice, Drobnica, Souri, and Oblica. The variability in squalene concentration may be attributed not only to the different cultivars, but also to the degree of ripeness of the fruit, agroclimatic conditions, and growing conditions [28,40,41]. Specifically, differences in genetic makeup have been shown to impact a cultivar’s capability to synthesize various compounds and enzymes, leading to qualitative and quantitative changes in the oil, such as varying levels of tocopherols and squalene [42,43].

Recently, the development of new olive cultivars that produce virgin olive oil with improved nutritional quality, including higher squalene content, has become a key goal in olive breeding programs. To achieve this, it is necessary to identify molecular markers associated with high squalene content in virgin olive oil. However, there is still limited knowledge on the genetic control of variability among olive cultivars, and molecular studies on squalene metabolism in olive fruit are lacking [27].

Extraction and refining processes lead to a notable decrease in squalene content. In particular, bleaching causes about 3% of squalene to isomerize due to the elevated temperatures and the presence of adsorbents acting as catalysts. This results in the formation of trans-isomers, which serve as a marker for distinguishing refined olive oil from virgin olive oil [39].

#### 3.3.2. Squalene in Olive Oil By-Products

While olive oil is the primary reason for cultivating olive trees, the squalene content found in olive by-products has increased their value and contributed to expanding the economic benefits of this plant while also reducing waste [28]. Sources of squalene related to the olive industry include leaves, pomace, olive tree bark, and overall olive oil deodorizer distillates [44].

The circular economy emphasizes sustainability by minimizing waste and maximizing the use of resources [45]. The extraction of squalene from olive by-products is a strong example of this approach. Olive oil production generates large amounts of by-products like olive pomace and leaves, which were traditionally seen as waste or used for low-value purposes. However, by extracting squalene, these by-products are transformed into valuable resources with high market demand. Utilizing olive oil by-products to extract squalene allows the olive industry to significantly reduce its environmental impact, improve resource efficiency, and open up new economic opportunities. By turning what was once considered waste into a valuable product, the industry aligns itself with sustainability goals while providing a greener, more ethical alternative to traditional squalene sources. This shift supports both ecological conservation and economic resilience within the olive oil industry. 

Due to its higher vapor pressure compared to most other substances in oil, squalene can be easily recovered from oil deodorizer distillates. Depending on the degree of vacuum and temperature applied during deodorization, the amount of squalene obtainable from distillates varies between 10 and 30% [46]. Squalene yields comprised between 224 and 452 g/kg from 10 different olive oil deodorizer distillates are documented [47]. 

Olive pomace is a by-product of olive oil production, consisting of the solid remains after the extraction of olive oil. This includes the olive skins, pulp, seeds, and stems. Despite being considered waste, olive pomace contains valuable compounds, including squalene and other minor bioactive compounds such as polyphenols [48]. Olive pomace is processed in refineries to extract the remaining pomace olive oil and refine it. To increase squalene yield, supercritical fluid extraction, ultrasounds, and molecular distillation have been applied [28]. The application of hydrothermal treatment of olive pomace led to a product enriched in minor components with functional activities. The final treated solid had an increase in oil yield by up to 97%, whereas squalene yield increased by up to 43% [49].

Olive leaves are a plentiful by-product of the olive industry, produced during the pruning of olive trees and the harvesting and processing of olives. Historically seen as waste, olive leaves have garnered attention for their potential applications thanks to their high content of bioactive compounds. Few studies deal with the chemical composition of olive leaves. Different compounds such as α-tocopherol, β-carotene, and β-sitosterol, among others, have been identified in olive leaf hexane extract [50], along with the presence of 38–152 mg/kg of squalene.

Similarly, other olive tree agro-industrial by-products such as olive bark are primarily generated from pruning. In Italy, it was estimated to be 1.7 tons/ha for olive trees [51]. Issaoui et al. [52] determined triterpenes and aliphatic hydrocarbons through supercritical fluid extraction (SFE) and gas chromatography (GC) coupled with mass spectrometry (MS), finding significant bioactive amounts, including squalene, in both olive leaves and tree bark.

### 3.4. Extraction and Purification of Squalene from Olive Oils and Olive By-Products

Various studies have applied pilot and industrial purification or extraction techniques to obtain squalene from alternative sources such as olive bark and olive oil by-products (Table 1).

Efficient extraction of squalene from residual pomace can be achieved through the use of ultrasound and molecular distillation, with yields reaching up to 740 g/kg [53]. In contrast, the squalene concentrations obtained from conventional extractions were much lower [50,53].

As previously reported, olive oil deodorizer distillates are likely one of the most concentrated squalene preparations. These by-products are obtained during the refining process of olive oil, specifically during the deodorization step, which aims to remove volatile compounds that may affect the taste and smell of the final olive oil product. By using supercritical carbon dioxide extraction on these distillates, the recovery yield can be increased and high-purity squalene can be attained (Table 1), making it suitable for pharmaceutical or cosmetic industries [28]. This is an environmentally friendly extraction method where no solvent is left after the extraction of the final product. In addition to the olive oil deodorizer distillates, the method was also studied for squalene extraction from olive leaves, olive bark [54,55], olive cake [56], and low-quality or lampante olive oils [57,58]. However, its main disadvantages are the energy requirements and economic investments. [50]. Pressurized acidic esterification in a closed system with vacuum distillation was also applied for squalene recovery from olive oil deodorizer distillates [59]. This method achieved a yield of 117 g/kg, proving to be a sustainable alternative to supercritical carbon dioxide extraction. A high recovery (76%) of high-purity (95%) squalene was obtained using a single-step isolation technique (centrifugal partition chromatography) [55], providing increased load capacity and better recovery compared to other extraction methods. In comparison to countercurrent supercritical carbon dioxide extraction, this method allows for higher purity and can be quick and efficient on an industrial scale.

Although none of the extractions presented in Table 1 have been studied on a real industrial scale, only on a laboratory or pilot scale, they could still be efficient and suitable methods for extracting squalene from olive oils and olive by-products for the industry.

While squalene itself does not pose a safety issue when produced on an industrial scale, the by-products and waste generated from extraction processes, especially those involving toxic chemicals (such as Soxhlet extraction, ultrasound-assisted extraction, and pressurized acidic esterification) require proper management. Without adequate handling, these by-products can lead to harmful emissions or leaks, causing significant damage to ecosystems. In contrast, environmentally friendly methods like supercritical fluid extraction (Table 1) and molecular distillation minimize the use of solvents, reducing the overall ecological footprint of squalene production [30]. Although extraction methods can be made safer, special attention must be given to the storage and transportation of squalene, as improper handling could result in the degradation of the product and the formation of harmful by-products, potentially impacting its safety and raising additional environmental concerns [60].

**Table 1:** Methods of extraction and purification of squalene from olive oils and by-products.

Method of Extraction	Principles, Advantages, and Disadvantages	Matrix and Yield	Ref.
Soxhlet extraction by hexane, isopropanol, and their mixture	Continuous extraction with hot organic solvents. Advantages: simple. Disadvantages: large amounts of organic solvents need it, and long extraction time.	Olive pomace: 5.217 mg/g Olive leaves: 0.038–0.152 mg/g	[50,53]
Ultrasound-assisted extraction	Ultrasonic waves create small air bubbles or cavities that increase temperature and pressure, resulting in improved penetration of the solvent and mass transfer. Advantages: It reduces the amount of the solvent and extraction time. It requires less energy compared to conventional extractions. Disadvantages: An additional step such as concentration and evaporation is required.	Olive pomace: 830 mg/mL	[53]
Pressurized acidic esterification in a closed system with vacuum distillation	An esterification reaction is conducted in a closed batch stirred reactor containing all the reactants (i.e., methanol, sulfuric acid, olive oil). Following this, distillation under vacuum (0.5 torr) is performed. Advantages: It improves the separation and quality of the final products. Disadvantages: High operating costs.	Olive oil deodorizer distillates: 117 mg/g	[59]
Molecular distillation	Type of short-path vacuum distillation involving low-temperature treatment as a result of vacuum application. The vacuum pressure is extremely low (0.01 torr or below). Advantages: No toxic solvents are needed. Reduce thermal decomposition. Disadvantages: Long times and high cost.	Olive pomace oil: 3700–10,000 mg/kg	[61,62]
Supercritical fluid extraction	The characteristics of supercritical fluid (usually CO_2_) such as gas-like diffusion, surface tension, viscosity, liquid-like density, and solvation power improve the solvent’s penetration and mass transfer. Advantages: It reduces the amount of the solvent and extraction time. It enhances the extraction yield. The extraction and concentration can be carried out in one step. Disadvantages: High instrumentation cost.	Olive oil deodorizer distillates: 64–82% yield in squalene (up to 92% purity) *Lampante* olive oil: 0.14 g/kg (90% recovery) Olive cake or pomace: 3450–5710 mg/100 g Olive leaves: 32% air Olive tree bark: 11% air	[52,54,56,57,58,63,64,65,66]
Centrifugal partition chromatography	Chromatographic pilot and industrial purification technique using liquid mobile and stationary phase prevented from moving by centrifugal force. Advantages: It has a high loading capacity which allows it to scale up. It enhances the extraction yield. Disadvantages: The efficiency of separation is dependent on different parameters (method, sample and solvent properties, parameters of instrumentation).	Olive oil deodorizer distillates: 19% yield (recovery 76%)	[55]

### 3.5. Squalene as a Dietary Supplement and Ingredient in Functional Foods

The food industry is increasingly exploring ways to incorporate squalene into various products, ranging from dietary supplements to fortified foods, in response to the growing consumer demand for wellness-oriented options [32]. As a nutraceutical supplement, squalene is used to enhance overall health and well-being. In addition to its role as a food additive, squalene also helps prevent oxidation in edible oils. It hinders the degradation of α-tocopherol during the photooxidation of olive oil and exhibits concentration-dependent antioxidant activity during the thermal oxidation process of oil [67].

The global market size of squalene has been growing significantly due to its increasing demand in various industries including cosmetics, pharmaceuticals, and food. It was valued at USD 141 million in 2022 and is projected to reach USD 202 million by 2028 [68].

Squalene can be included in capsules or tablets as a dietary supplement to deliver its health benefits directly. Orally administered squalene is well absorbed (60–85%) and distributed to various tissues. Therefore, it has been extensively used as a carrier/adjuvant in therapeutic applications, including vaccines [19,26]. The most common forms of squalene supplements are capsules and softgels, which offer a convenient and precise dosage. Mixing squalene with cooking oils or fish oils, like liquid supplement, provides an easy way for consumers to incorporate this beneficial compound into their daily diet. Additionally, squalene can be formulated as a powder, which can be added to health drinks, smoothies, and juices to enhance their benefits, especially for their antioxidant and anti-inflammatory properties [26,69].

Due to its vulnerability to oxidation, strategies for squalene encapsulation in food matrices are necessary. Kumar Lekshmi et al. [70] successfully investigated the effectiveness of chitosan–whey protein as a wall material for squalene encapsulation in functional foods. Later, the same authors found optimal performance for a blend of maltodextrin and whey protein [71] and investigated the physical and quality attributes of muffins fortified with microencapsulated squalene [72].

Sponton et al. [73] encapsulated squalene to be added to a bread matrix using a conventional emulsion method, enhancing its stability. They used egg white protein nanoparticles as a surfactant, followed by freeze-drying to obtain squalene powder as an ingredient. The addition of 5.0% freeze-dried squalene powder did not impact the physical, textural, or sensory properties of the functional bread.

## 4. Analytical Determination of Squalene

The initial studies on determining squalene used a colorimetric method. This method involved starting color development (due to the formation of polycyclic derivatives) by adding an acidic solution, such as concentrated sulfuric acid, which would result in a pale-yellow color appearing after heating at 70 °C for 5 min. To intensify and stabilize the color, formaldehyde was added [74]. However, this method required a laborious drying stage because even small amounts of solvents could disrupt the color development. Furthermore, there was a risk of a positive reaction with other compounds containing double bonds, so the test needed to be conducted after a purification step.

The only official method for determining squalene in oils and fats is the one proposed in 1999 by the Association of Official Analytical Chemists (AOAC 943.04.1999) [75]. This method involves several steps to accurately quantify squalene, including sample saponification, extraction of non-saponifiable matter using large amounts of solvents, fractionation by column chromatography, and iodometric titration to take advantage of the presence of multiple double bonds in the squalene molecule.

Regulation (EC) 2104/2022 [76] on the characteristics of olive oil establishes limits on the contents of certain unsaponifiable components, and Regulation (EU) 2105/2022 [77] outlines the appropriate methods of analysis. Squalene does not have specific legal limits but it can be quantified alongside waxes and methyl and ethyl esters of fatty acids using the COI method T.20/Doc.No28 [78], which involves fractionation on a silica gel column followed by direct GC analysis without derivatization. By applying this method, squalene elutes around the middle of the chromatogram, between the methyl and ethyl esters and the waxes. Alternatively, squalene can be isolated from the unsaponifiable matter using the COI method T.20/Doc.No30 [79] for the GC determination of sterols and triterpene dialcohols. This method involves saponification with potassium hydroxide in an ethanolic solution, extraction of the unsaponifiable matter with ethyl ether, and squalene isolation from other compounds (i.e., triterpene and aliphatic alcohols, sterols, and triterpenic dialcohols, and free fatty acids) by thin-layer chromatography (TLC) on a basic silica gel plate.

Based on these premises, a key challenge in squalene quantification is the absence of a validated, standardized method specifically designed for squalene determination in olive oil and its by-products. This lack hampers efforts to enhance the valorization of squalene as a functional ingredient. Most current methodologies are designed to detect other oil components (such as triacylglycerols, sterols, or tocopherols), with squalene often being only co-determined as a secondary analyte. This gap in the methodology underscores the necessity of developing a specific method, validated and focused only on squalene, ensuring more accurate, reliable, and comparable measurements, particularly for assessing its nutritional and commercial value.

**Figure 2:**
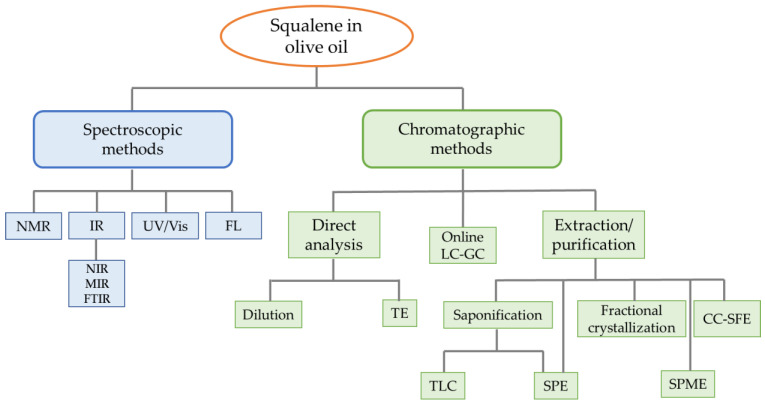
Scheme of the main analytical approaches, classified into spectroscopic and chromatographic methods, used for squalene analysis in olive oil samples. NMR: nuclear magnetic resonance spectroscopy; IR: infrared; NIR: near-infrared; MIR: mid-infrared; FTIR: Fourier transform infrared; UV/Vis: ultraviolet/visible; FL: fluorescence; TE: transesterification; LC-GC: liquid–gas chromatography; CC-SFE: counter-current supercritical fluid extraction; TLC: thin layer chromatography; SPE: solid phase extraction; SPME: solid phase microextraction.

Figure 2 reports a scheme of the main analytical approaches, classified into spectroscopic and chromatographic methods, used for squalene analysis in olive oil samples. Special attention is given to the different approaches for sample preparation before analytical determination, which is crucial, especially in chromatographic techniques.

### 4.1. Spectroscopic Methods

Spectroscopic methods, while generally less specific than chromatographic techniques, offer a simpler and more environmentally friendly alternative for determining squalene concentration in olive oil and by-products. These methods, which rely on the interaction of light with squalene molecules and are often integrated with chemometric techniques, have become more attractive due to their minimal sample preparation, faster analysis times, and avoidance of harmful solvents.

The application of nuclear magnetic resonance spectroscopy (NMR) to olive oil represents a reliable and rapid tool to verify various aspects of its adulteration, including undisclosed blends with cheaper oils and the mislabeling of cultivar and geographical origin. This is possible due to NMR’s ability to determine the olive oil metabolomic profile and quantify its constituents, including squalene [80]. For example, ^13^C-NMR without saponification, extraction, or fractionation was validated using pure squalene in triolein. It was then applied to determine squalene content in 24 olive oil samples from Corsica, offering a non-destructive and precise alternative [81]. Another example of how NMR can be used for quantification, even without expensive standard references or troublesome calibration curves, was provided by Rotondo et al. [82]. They validated an unreferenced and accurate quantification of squalene in vegetable oils through the analysis of 1H-NMR spectra and the standard addition method simply by diluting the sample in deuterated chloroform.

Near-infrared (NIR) and mid-infrared (MIR) spectroscopy, combined with chemometric data analysis, have emerged as promising methods for determining functional compounds in olive oil, including squalene [83]. The first attempt to use NIR spectroscopy to specifically measure squalene in olive oil was reported by Cayuela et al. [84] successfully classifying samples based on their squalene content.

The spectroscopic techniques of ultraviolet-visible (UV-Vis), fluorescence, and Fourier transform infrared (FTIR) were tested using partial least squares (PLS) calibration models for the quantification of squalene in amaranth seed oil [85]. Furthermore, fluorescence spectroscopy is especially effective in quantifying squalene in extra virgin olive oil within a range of 3.25 to 12.54 g/kg, without the need for any destructive processes or sample pretreatment [86].

Although spectroscopic analysis has many advantages compared to chromatographic techniques, including speed, minimal sample preparation, and environmental friendliness, it also has drawbacks. In particular, spectroscopic methods may lack sensitivity and specificity, making it difficult to accurately detect and quantify squalene, especially when it is present in low concentrations or in the presence of other similar compounds that can interfere with the signal. Vegetable oils are complex mixtures that contain various components such as fatty acids, phenols, and other lipophilic substances. These components can interfere with the spectroscopic signal, leading to the inaccurate quantification of squalene. The overlapping absorption or emission spectra can be particularly problematic, complicating the interpretation of results. Additionally, data generated from spectroscopic techniques, especially when combined with chemometric techniques, can be complex and require advanced statistical tools for accurate interpretation, making it not readily accessible or understood by all users. Furthermore, high-quality spectroscopic instruments are expensive, limiting their accessibility for routine analysis.

### 4.2. Chromatographic Methods

For a more precise and efficient quantification of squalene, chromatographic methods are commonly used. These methods can be further subdivided based on the complexity involved in the sample preparation step (Figure 2). Direct analysis can be conducted by skipping extraction and purification steps, instead opting for simple dilutions of the oil in a suitable solvent or derivatization before analytical determination. On the other hand, there are available methods that use extensive sample preparation to isolate the compound of interest from the matrix. This includes the removal of triglycerides and the fractionation of the unsaponifiable compounds into several classes, for example, through solid phase extraction (SPE) with or without prior saponification, or through fractional crystallization, solid phase microextraction (SPME), and counter-current (CC)-SFE. Despite the advantage of greater accuracy in quantification, pre-treatment usually involves relatively high volumes of organic solvent, high capital costs, and long processing times, and can result in a significant loss of squalene [71,72]. The online liquid–gas chromatographic (LC-GC) method can be positioned between these two groups of approaches. In fact, in this method, the sample can be directly injected into the instrument, and the presence of the LC column enables the retention of triglycerides, thus preventing their transfer to the GC column.

Squalene quantification is typically performed using high-performance liquid chromatography (HPLC) coupled with an ultraviolet (UV) detector, diode array detector (DAD), refractive index detector (RID), or MS detector or by GC coupled with a flame ionization detector (FID) or MS detector [71].

Details on sample preparation, analysis time, and analytical performance are provided in Table 2.

#### 4.2.1. Direct Injection After Sample Dilution

A rapid and simple screening method was recently proposed by Hayakawa et al. [88], focusing on squalene (and tyrosol) quantification, which can serve as an effective marker for detecting adulterated extra virgin olive oils. In this method, the oil samples undergo a simple pretreatment, where they are diluted (1:20) with 2-propanol and the squalene content is determined using HPLC-UV. The best elution performance was obtained using columns filled with a packing of octadecylsilyl groups (ODS) and gradient elution with acetonitrile and 2-propanol (starting from 10% to 50%), providing a better separation of triglycerols. Despite the absence of a purification step before chromatographic analysis, very low limits of detection (LOD) and quantification (LOQ) are documented (0.042 and 0.126 mg/L, respectively). Additionally, despite the obvious elution of triglycerides, no issues were reported from the LC column being washed with acetonitrile/2-propanol (1:1, *v*/*v*) for 25 min after the injection of each sample.

Squalene can be analyzed using a RID [87]. Also in this case, a simple sample dilution (acetone/acetonitrile 70:30, *v*/*v*) and centrifugation are the only steps required before HPLC analysis. The triglycerides, not removed during sample preparation, elute later. While the RID method may not be as sensitive as some alternatives, its combination with MS for identification and the use of the standard addition method for quantification make it a viable approach for analyzing squalene in olive oil. The LOQ of 6.3 mg/L is appropriate given squalene’s relatively high occurrence in olive oil, ensuring that this method can effectively quantify its presence within the typical concentration range.

#### 4.2.2. Transesterification

The transmethylation reaction used for the analysis of fatty acid methyl esters (FAMEs) before direct GC analysis [92] is an alternative method to analyze squalene without the need for sample extraction or purification. Specifically, 15 mg of oil is dissolved in 1 mL of hexane and mixed with 0.1 mL of methanolic potassium hydroxide (2 M). The upper phase that separates from the mixture can be directly injected into GC. Base-catalyzed transmethylation also has the advantage of proceeding very rapidly (approximately 2 min) at room temperature. Approximately 10 min are needed for sample preparation, and the total GC run lasts about 5 min. This represents the latest evolution of the transmethylation approach for investigating squalene, previously utilized by other authors [90,91].

Furthermore, alkaline transesterification followed by hydrogenation using platinum dioxide as a catalyst allows for the conversion of squalene into squalane (a fully saturated hydrocarbon), the target analyte, which can then be analyzed in GC along with fully saturated methyl esters [93,100]. A correction factor was required to adjust the FID response because hydrocarbons have a higher response compared to the methyl esters of fatty acids.

#### 4.2.3. Online LC-GC

Online LC-GC is an innovative technique that integrates sample preparation with chromatographic analysis, offering a streamlined alternative to traditional, labor-intensive sample preparation methods. This approach has the advantage of allowing for both the direct analysis of squalene and the simultaneous analysis of other fractions of interest in the unsaponifiable matter of edible oils by avoiding saponification. By applying the official method (ISO 20122:2024) [101] for the determination of saturated (MOSH) and aromatic (MOAH) hydrocarbons in vegetable fats and oils, without sample pre-enrichment by saponification (not needed since squalene is present at relatively high amount) and without epoxidation (used to eliminate interference by olefins when analyzing the MOAH), the squalene peak elutes in the MOAH fraction. The mobile phase used to elute the MOAH fraction is a mixture of hexane and dichloromethane in a ratio of 70:30 (*v/v*). After transferring the MOAH fraction, the LC column needs to be backflushed with dichloromethane to eliminate triglycerides remaining in the column and reconditioned with hexane before the next run.

**Figure 3:**
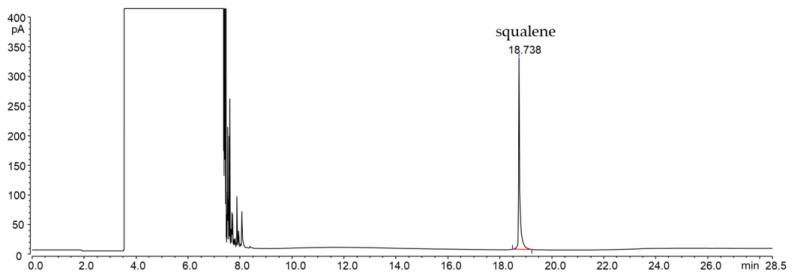
LC-GC-FID chromatogram (MOAH fraction) of an extra virgin olive oil diluted 1:200 in hexane.

Figure 3 shows the LC-GC chromatograms of the MOAH fraction of two different EVOOs analyzed, after simple sample dilution, by online LC-GC in our laboratory, according to the chromatographic conditions described by Menegoz et al. [102].

The reverse-phase (RP)LC-GC method has also been proposed for the analysis of free sterols, tocopherols, and squalene in only one chromatographic run by direct injection, without the need for prior enrichment of the sample, reaching a LOD of 0.3 mg/kg [94]. RPLC-GC has the advantage that triglycerides elute before minor compounds and squalene, and hence there is no need to backflush the LC column.

#### 4.2.4. Saponification

In olive oil, the chemical composition includes a saponifiable fraction, which constitutes 98–99% of the total weight, composed mainly of triglycerides, and an unsaponifiable fraction, which represents 0.5–2% of the total weight. The unsaponifiable fraction contains several bioactive components, such as squalene, β-carotene, tocopherols, and tocotrienols [103].

Traditional sample preparation involves a saponification step, adopted in most of the GC methods, which involves the hydrolysis of the saponifiable fraction, breaking down triglycerides into their constituent fatty acids and glycerol [104]. This allows for the separation of squalene and other unsaponifiable components from the bulk of the oil matrix, enabling their subsequent quantification. Saponification is typically performed using ethanolic potassium hydroxide (KOH) at different concentrations and temperature–time combinations. After saponification, unsaponifiable compounds can be extracted using organic solvents, followed by phase separation via centrifugation or using a separation funnel. Fractionation of unsaponifiables is carried out using SPE, TLC, or liquid–liquid extraction. For GC analysis, squalene is often analyzed directly without derivatization, although derivatization can be applied for other compounds or specific purposes [39,95].

Saponification without fractionation is proposed for the direct determination of sterols, aliphatic alcohols, squalene, and tocopherol contents using GC [105]. In this method, the oil is mixed with ethanolic potassium hydroxide and subjected to saponification at 40 °C for 40 min. After washing with water, the solution is transferred to a decanting funnel with the aid of hexane and allowed to separate. The hexane extract is then washed repeatedly with water, passed through anhydrous sodium sulfate, and evaporated to dryness. Following silylation with hexamethyldisilazane, trimethylchlorosilane and pyridine (2:1:10, *v*/*v*/*v*) and dissolution in chloroform, the supernatant is injected to the GC for analysis.

#### 4.2.5. Solid Phase Extraction (SPE)

SPE on a silica cartridge was also used to isolate and elute squalene after dissolving the extra virgin olive oil in hexane [36,96,106]. Elution from the silica column required approximately 85 mL of a mixture of hexane/diethyl ether 95:5 (*v*/*v*). It followed GC-FID analysis, which was completed in less than 10 min [106]. Alternatively, the eluate from the silica cartridge in approximately 10 mL of hexane can be analyzed by HPLC-UV with improved selectivity at 217 nm compared to 208 nm, as other compounds eluted before squalene were not detected. Squalene elution is obtained in less than 1 min when using ultra high-performance liquid chromatography (UHPLC) [96] and within 10 min using a traditional HPLC [36].

#### 4.2.6. Fractional Crystallization

To streamline the sample preparation process, fractional crystallization was suggested [97,98], followed by HPLC analysis. A methanol/acetone mixture (7:3, *v*/*v*) is used to dissolve squalene, and the different solubilities of components aid in fractionation. Specifically, squalene tends to remain in the organic phase, while other components of olive oil that are not soluble precipitate out. This process helps to remove the majority of triglycerides, especially those with an equivalent carbon number greater than 44. Additionally, this method allows for the separation of interferences with high melting points, such as cholesterol, stigmasterol, and brassicasterol, that can coelute with squalene when present in high amounts, as seen in olive oil. Failing to separate them in advance would lead to prolonged analysis times, requiring more than 1 h to ensure the complete elution of potential interferences by backflushing the LC column after each analysis. However, despite its effectiveness in sample handling, long freezing times of 30 h are necessary to enable proper fractionation, even if constant supervision by the analyst is not required. An additional benefit of this approach is the increase in column life, as it is not burdened with the more saturated species expected at the end of the chromatographic run.

Fractional crystallization was also used for squalene determination in olive oil samples using GC-FID [99]. Only a small quantity of organic solvent (10 mL of methanol/acetone 7:3, *v*/*v* for the fractionation phase and 2.5 mL of heptane to reconstitute the residue before injection into GC) was needed. LOD (19 mg/kg) and LOQ values (63 mg/kg) were significantly lower than usual squalene concentration levels in olive oils.

After triglyceride precipitation, liquid–liquid extraction can be used to further refine the extraction of squalene and remove additional interferences such as sterols. To achieve this, a non-polar solvent like heptane or hexane can be used. Squalene, being non-polar, preferentially partitions into the organic (non-polar) phase, whereas polar contaminants like some sterols and minor components will remain in the aqueous phase. The two phases are then separated. The organic phase, now containing mostly squalene and minimal contaminants, is collected, while the aqueous phase is discarded.

#### 4.2.7. Solid Phase Microextraction (SPME)

A solventless extraction technique, headspace (HS)-SPME, was used for squalene determination in olive oil samples by GC-MS [99]. This approach requires a more expensive instrumental set-up but has the distinctive advantages of being very simple (no sample pre-treatment), relatively fast (equilibration of the headspace at 80 °C for 60 min, SPME exposure for 30 min, and total chromatographic acquisition of about 45 min), solventless, and producing the best performance in terms of LOQ (8 mg/kg), even though it is not necessary when analyzing EVOO samples.

#### 4.2.8. Supercritical Fluid Extraction (SFE)

This approach has certainly been most widely used for the extraction of squalene from by-products of the oil supply chain, as described below. A process based on semi-CC-SFE was optimized to concentrate minor components, including squalene, from olive oil, while preserving its functional properties. This method enhances efficiency by continuously feeding the extractant (supercritical CO_2_) and the oil matrix into a column in opposite directions, maximizing contact between the two phases. The optimized conditions, found through a surface response methodology, maximize squalene recovery, achieving approximately 90% [57]. Despite the numerous advantages this approach offers, such as being eco-friendly, safe, selective, and highly efficient, drawbacks such as high initial costs, the need for specialized technical knowledge, and energy-intensive processes make this application difficult for routine laboratory analysis.

### 4.3. Analytical Determination of Squalene in Olive Processing By-Products

The analytical determination of squalene in olive processing by-products is a challenging task due to the complex nature of the matrix involved. The same methods used on a laboratory or pilot scale to obtain squalene from different olive oil by-products (see Table 1) could be used for analytical purposes. In 2015, Popa et al. [30] published a comprehensive review of analytical techniques for determining squalene from plant sources, including some by-products from the olive oil supply chain. The commonly used analytical methods for determining squalene in olive processing by-products are GC-FID [50,54,61,62,63,64] or are coupled with MS [56,64,65] or HPLC-DAD [53,57,58,59]. The methods used to detect squalene in olive processing by-products are the same as those used for olive oil. However, sample preparation and extraction methods differ when dealing with solid matrices. In particular, leaves and bark trees are dried, crushed, and extracted with hexane [50,65], or directly loaded in the supercritical reactor [52]. Samples with a high water content, such as patè olive cakes, are freeze-dried and ground before the extraction [56].

The extraction methods can be either conventional (i.e., Soxhlet extraction) or non-conventional (i.e., SFE, ultrasound extraction). A comparison between ultrasound-assisted extraction and conventional Soxhlet extraction revealed that the former gave higher levels of squalene in olive pomace oil when hexane was used as a solvent, whereas a mixture of hexane and isopropanol resulted in similar concentrations [53], indicating that solvent composition can influence the relative effectiveness of these two extraction techniques.

To avoid toxic solvents and utilize a greener extraction technology, supercritical fluid extraction was used to extract squalene from olive oil deodorizer distillates, *lampante* olive oil, olive cake or pomace, olive leaves, and olive tree [52,54,56,57,58,63,64,65,66]. Supercritical carbon dioxide is used as a solvent in most cases, with the possible addition of a co-solvent. Carbon dioxide is chosen for its low price, inertness, high volatility, and nontoxicity. This method can be efficient, resulting in high-purity squalene extraction [30].

## 5. Conclusions and Future Perspectives

The analysis of squalene in olive oil and its by-products is crucial due to its high economic and nutritional value. Squalene, a bioactive compound with antioxidant and skin-protective properties, plays a critical role in the health benefits associated with olive oil consumption. Knowing the precise concentration of squalene in olive oil and its derivatives is essential for determining the quality and value of these products, as well as for optimizing their use in the food, cosmetic, and pharmaceutical industries.

Furthermore, understanding the squalene content in by-products of olive oil processing opens up opportunities to revitalize these by-products. By extracting squalene and other bioactive molecules, the sustainability of olive oil production can be enhanced, adding value to what would otherwise be considered waste. This allows for the recovery of compounds with high market demand, promoting a circular economy within the olive oil industry.

However, a significant limitation in the current landscape is the lack of official methods for the quantification of squalene. Different research approaches and analytical techniques provide varying levels of accuracy and sensitivity, but no standardized protocol has been established. The absence of a unified, official method hampers the comparability of results across studies and industries. Thus, there is an urgent need for the development of a reliable, standardized method for squalene quantification to ensure consistency and accuracy in determining the value of olive oil and its by-products. Developing a consistent, cost-effective, and environmentally responsible method will further enhance the value of squalene in the global market, making it easier for industries to recover this important compound and promote its health benefits on a larger scale.

Additionally, the development of a method for squalene analysis should prioritize economic sustainability and low environmental impact. This includes creating an extraction and recovery method that is efficient and can be easily applied on an industrial scale for squalene from both primary products and food by-products. A scalable, eco-friendly approach would not only reduce the environmental footprint of olive oil production but also make it economically viable for widespread use in various industries. By focusing on sustainability, the olive oil industry can maximize the benefits of squalene extraction while adhering to modern environmental and economic standards, ensuring long-term growth and innovation.

## Figures and Tables

**Table 2:** Details on sample preparation, analysis time, and analytical performance of some chromatographic methods used for analyzing squalene in olive oils. LOD: limit of detection; LOQ: limit of quantification; RSD: relative standard deviation; SFE: supercritical fluid extraction.

	Sample Preparation		Analytical Determination			Ref.
	Extraction	Additional Treatment	System	Time of Analysis	Performance	
**Sample dilution**	100 mg of oil diluted with acetone/acetonitrile 70:30 (*v*/*v*) to 2 mL	Centrifugation.	HPLC-RID	Elution of squalene in 7 min	LOQ: 6.3 mg/L	[87]
	Oil is diluted with 2-propanol in a ratio of 1:20		HPLC-UV (215 nm)	Total run time of 60 min	Range: 0.126–300 mg/L; LOD: 0.042 mg/L; LOQ: 0.126 mg/L; RSD <2%; Recovery: 95–105%	[88]
	100 µL of oil dissolved in 900 µL 2-propanol	Filtration (0.22 µm).	HPLC-DAD (210 nm)	Elution of squalene in 16.5 min	Range: 20–600 mg/L; LOD: 3.70 mg/L; LOQ: 11.1 mg/L; RSD: <8.4%; Recovery: 92–109%	[89]
**Trasesterification**	200 mg of oil dissolved in 5 mL of hexane	Addition of 1 mL of methanolic KOH (2 N) and vortex for 1 min. After a rest of 10 min, the hexane phase is transferred and washed with 2 × 4 mL of ethanol/water 1:1 (*v*/*v*).	GC-FID	Total run time of 15 min	Range: 5–25 g/kg; RSD: <2%	[90]
	100 mg lipid dissolved in 1 mL of t-BME	Addition of 0.5 mL of sodium methoxide in methanol (0.2 M) and 0.1 mL of H_2_SO_4_ (0.5 M) to stop the reaction. Addition of distilled water (~1.5 mL), centrifugation, collection of the upper phase, and drying over anhydrous Na_2_SO_4_. The upper phase was then transferred to a final test tube, the solvent was evaporated, and the sample was redissolved in hexane.	GC-FID	Total run time of 30 min	Range 0.5–20 g/L; LOD: 0.4 g/L; LOQ: 1.3 g/L; RSD: 2.7%	[91]
	15 mg of oil in 1 mL of hexane	Addition of 0.1 mL of methanolic KOH (2 M), centrifugation, and direct analysis of the upper phase.	GC-FID/MS	Total run time of 5 min	Range: 10–500 mg/L; LOD: 0.007%; LOQ: 0.022%; RSD: 1.1%; Recovery: 95.2–97.4%	[92]
	Oil	Alkali transesterification and extraction in chloroform, followed by hydrogenation with PtO_2._	GC-FID	Total run time of 30 min		[93]
**Online LC-GC**	Oil	Filtration (0.22 µm).	(RP)LC-GC-FID	Total run time of approximately 50 min	LOD: 0.3 mg/kg; RSD: 6%	[94]
**Saponification**	0.1–0.2 g of oil added with 2 mL of ethanol	Saponification at 56 °C for 10 min with KOH (0.6 mL and 50% *w*/*v*), addition of 1mL of deionized distilled water with 1% NaCl and hexane (4 × 2 mL), and centrifugation. The supernatant hexane phase was transferred and dried under nitrogen flow at 40 °C. Silylation at 30 °C for 10 min with hexamethyldisilazane/trimethylchlorosylane/pyridine (260 mL, 3:1:9, *v*/*v*). Centrifugation and separation of the upper phase.	GC-FID	Time of analysis was reduced to approximately 5 h for every six samples compared to approximately 20 h of traditional methods	LOQ: 160 mg/kg; RSD: 1%; Recovery: 93–98%	[95]
**SPE**	120 mg of oil dissolved in 0.5 mL of hexane	SPE silica (1 g) cartridge conditioned with 6 mL of hexane and squalene was eluted with 10 mL of hexane. Evaporation under reduced pressure and dissolution in the HPLC eluent.	HPLC-UV (208 nm)	Elution of squalene in less than 12 min	LOD: 0.62 g/L; LOQ: 0.78 g/L; Recovery: 88–85%	[36]
	120 mg of oil dissolved in 0.6 mL of hexane	SPE silica (500 mg) cartridge conditioned with 5 mL of hexane and squalene was eluted with 10 mL of hexane. Evaporation under vacuum and dissolution in 1 mL of mobile phase.	UHPLC/PDA (217 nm)	Elution of squalene in less than 1 min	Range 50–500 mg/L; LOD: 0.3 mg/L; LOQ: 1.0 mg/L; RSD: <3.38%; Recovery: 91.9–96.3%	[96]
**Fractional Crystallization**	500 mg of oil vortexed with 20 mL methanol/acetone 7:3 (*v*/*v*)	Fractional crystallization at –22 °C for 24 h. The supernatant was filtered through a filter paper, the solvent was evaporated under vacuum at 40 °C and the residue dissolved in acetone (5 mL).	HPLC-UV (208 nm)	Elution of squalene in 11 min	Range:20–400 mg/kg; LOD: 23 mg/kg; LOQ: 79 mg/kg; RSD: 3.76%; Recovery: 85.1–92.5%	[97]
	100 mg of oil mixed with methanol/acetone 7:3 (*v*/*v*)	Fractional crystallization at –20 °C for 30 h. The supernatant was filtered, the solvent was evaporated to dryness, and the residue was dissolved in mobile phase.	HPLC-UV (195 nm + spectrum 190–400 nm)	Total run time of 16 min (>1 h for complex matrix)	Range: 0.1–40 mg/L; LOD: 0.04 mg/L; RSD: 0.9%; Recovery: 89.7–96%	[98]
	125 mg olive oil vortexed with 10 mL methanol/acetonitrile 7:3 (*v*/*v*)	Fractional crystallization at –20 °C × 24 h. Centrifugation, evaporation under vacuum, and dissolution in heptane (2.5 mL).	GC-FID	Total run time of 40 min	Range: 1–10 g/kg; LOD: 0.019 g/kg; LOQ: 0.063 g/kg; RSD: <7%; Recovery 68–72%	[99]
**SPME**	2 g of oil in a 4 mL vial hermetically closed	Conditioning at 80 °C for 60 min and exposure of PDMS fiber in the headspace for 30 min.	GC-MS	Elution of squalene in less than 15 min	LOD: 3 mg/kg; LOQ: 8 mg/kg; RSD: <6%; Recovery: 98–101%	
**SFE**		Countercurrent SFE at 234 bar, 35 °C, CO_2_ flow of 2000 mL/h, and sample flow rate of 82 mL/h.	HPLC-UV (225 nm)	Total run time of 30 min	Recovery: 90%	[57]

## Data Availability

Data are contained within the article.
